# An orexin-sensitive subpopulation of layer 6 neurons regulates cortical excitability and anxiety behaviour

**DOI:** 10.1038/s41398-025-03350-2

**Published:** 2025-04-14

**Authors:** Fernando Messore, Rajeevan Narayanan Therpurakal, Jean-Philippe Dufour, Anna Hoerder-Suabedissen, Luiz Guidi, Kim Korrell, Marissa Mueller, Mohammed Abuelem, Armin Lak, David M. Bannerman, Edward O. Mann, Zoltán Molnár

**Affiliations:** 1 Department of Physiology, Anatomy and Genetics University of Oxford, Oxford, USA; 2 National Institute of Mental Health, National Institutes of Health, Bethesda, MD USA; 3 Department of Experimental Psychology, University of Oxford, Oxford, UK; 4 Present Address: Department of Neurology, Medical Faculty and University Hospital Düsseldorf, Heinrich Heine University, Düsseldorf, Germany

**Keywords:** Molecular neuroscience, Psychiatric disorders

## Abstract

Cortical layer 6 neurons are the only projection neuron population in the cortical mantle known to electrophysiologically respond to orexin—a neuropeptide involved in cortical arousal and emotive behaviour. These neurons exhibit extensive intercortical and thalamic projections, yet the exact mechanisms underlying these responses are not fully understood. We hypothesize that cortical circuits activated by orexin sensitive L6 neurons in the medial prefrontal cortex (mPFC) are responsible for detecting salient features of sensory stimuli and are therefore involved in regulating emotional states. Here, we show that Drd1a-Cre+ neurons in the mPFC are selectively sensitive to orexin and gate the activation of the prefrontal network in vivo. Moreover, we demonstrated that chronically “silencing” this subpopulation of L6 neurons (Drd1a-Cre^+/+^:Snap25^fl/fl^) across the cortical mantle from birth abolishes the orexin-induced prefrontal activation. Consequently, the chronic silencing of these neurons had strong anxiolytic effects on several anxiety-related behavioural paradigms, indicating that orexin-responsive L6 neurons modulate emotional states and may be a substrate for anxiety regulation.

## Introduction

Layer 6 (L6) is the innermost layer of the cerebral cortex, which contains diverse types of projection neurons that likely mediate distinct circuit functions through both intra- and interhemispheric cortical connections, as well as feedforward or feedback projections to thalamus [1–3]. This layer contains the only cortical neurons known to exhibit direct electrophysiological response to orexin [4–6], a neuropeptide released from neurons in lateral hypothalamus and perifornical area, which has been implicated in regulating arousal, wakefulness and feeding behaviour [7]. The orexinergic system can regulate behavioural state via effects on subcortical targets, including other hypothalamic nuclei [8] and arousal centres, such as the noradrenergic neurons in the locus coeruleus [9]. While the role of direct orexinergic projections to cortical L6 remain less well understood, they may contribute to cortical arousal and stress responses [10]. In sensorimotor neocortex, it has been shown that a Cre-driver mouse line targeting neurons expressing dopamine receptor 1a (Drd1a-Cre) selectively labels a subpopulation of L6 projection neurons, which are concentrated close to the white matter, termed layer 6b (L6b), and send ascending projections to layer 1 and feedforward projections to higher-order thalamus [11–16]. Recordings in somatosensory cortex have revealed that it is this subpopulation of L6 neurons that display selective sensitivity to orexin. Moreover, optogenetic activation of these Drd1a-Cre+ neurons is sufficient to trigger awake-like network states in the neocortex of sleep-deprived mice [17] and silencing them blunts the effects of orexin on wake-related theta activity in freely moving animals [18], consistent with a role for cortical Drd1a-Cre+ neurons in mediating an orexinergic modulation of cortical state.

Recent work suggests that the prefrontal cortex (PFC) is particularly involved in regulating behavioural responses to orexin [19–21]. However, it remains unclear whether the anatomical and physiological properties of the Drd1a-Cre+ neurons are conserved across higher association areas, to what extent Drd1a-Cre+ neurons mediate PFC orexin sensitivity, or what behavioural function this deep Drd1a-Cre+ PFC subcircuit might serve. Here, we systematically analyse the cellular properties of Drd1a-Cre+ neurons and their role in the activation of medial prefrontal cortex (mPFC). We evaluate orexin sensitivity by chronically silencing this subpopulation of L6 neurons (Drd1a-Cre+;Snap25^fl/fl^;Ai14) across the cortical mantle from birth. This results in decreased cortical response to Orexin receptor 2 (OX2R) agonists in the mPFC and decreased anxious behaviour in adult mice.

## Material and methods

### Animals

All animal experiments were approved by a local ethical review committee and conducted in accordance with personal and project licenses under the UK Animals (Scientific Procedures) Act (1986). Mice were housed in a temperature-controlled room under a 12 h light/12 h dark cycle, with free access to food and water, and pups were kept with their dam until weaning age at P21, or the experimental endpoint if earlier. A total of 64 adult mice were used in this study. A mix of male and female animals (n = 49 and n = 15 respectively) were used in these experiments. Distinct Cre-recombinase-expressing strains were used for each experiment. The specifics of the genotypes used in each experiment are explained in detail in the corresponding section. A complete description of the animals used, and their genotype is shown in Table S1.

### Immunohistochemistry

Adult mice were anesthetised and culled by intraperitoneal pentobarbitone overdose (Animalcare, XVD135) followed by exsanguination and perfusion fixation using 0.1 M phosphate buffered saline (PBS, pH = 7.4) and 4% formaldehyde (PFA diluted in PBS, Sigma Aldrich, F8775). Brains were dissected, post-fixed for 24 h in PFA, then stored in 0.1 M PBS with 0.05% sodium azide at 4 °C. Brains were embedded in 4.5% agarose, cut into 50 μm coronal sections using a vibrating microtome (VT1000S, Leica Systems, Wetzlar, Germany), and placed into 24-well plates (one section/500 μL/well). Tissues were washed 3×10 min in 0.1 MPBS, incubated in a blocking solution of 10% donkey serum (Sigma Aldrich, D9663) and 0.3% Triton^TM^ X-100 (Thermo Scientific^TM^, 85111) in 0.1M PBS (DS-10%;TX-0.3%;1XPBS) for 2 hr at room temperature (RT), then with primary antibodies Rb α Cplx3 (1:1000, Synaptic Systems 122302) and Ms α Tbr1 (1:500, ProteinTech 66564-1-LG) in DS-5%;TX-0.3%;1XPBS for 48 hr at 4 °C. Sections were washed 3×10 min, incubated with secondary antibodies Dk α Rb (488 nm, 1:500, Invitrogen A21206) and Dk α Ms (647 nm, 1:500, Invitrogen, A32787) in DS-5%;TX-0.3%;1XPBS for 2 hr at RT, washed 3 × 10 min in 0.1 M PBS, incubated with 1:1000 4′,6-diamidino-2- phenylindole (DAPI) for 30 min at RT, then washed 2 × 10 min in 0.1 M PBS. Tissues were mounted with 60 μL ProLong^TM^ Glass (Invitrogen^TM^, P36984) per slide.

### Imaging and analysis

Spinning-disk confocal microscopy (Olympus SpinSR SoRa) was conducted at 20X magnification, using cellSens (v4.1.1, Evident, Boston, MA, USA) to apply high-density focus maps with additional points as required. Images were pre-processed using a custom Fiji/ImageJ.ijm script (2.9.0 v1.54b). Coronal sections were imported with split channels at a downsampled factor of 4 (Series 3) followed by maximum-intensity z-stack projections, auto-brightness thresholding, and background subtraction. Outputs were imported into QuPath (v0.4.2) [22] where a custom Groovy script detected cells and exported two sets of images. The first contained.svg segmentations, which were re-formatted for Nutil compatibility [23] using another custom Fiji/ImageJ.ijm script (i.e., binary masks application, pixel inversion, and file type conversion to RGB.pngs). The second set of images were saved as RGB.pngs with 8 × downsampling for QuickNII compatibility (RRID:SCR_016854) [24]. Downsampled.pngs were formatted using QuickNII’s FileBuilder [24] for subsequent section alignment and Allen atlas registration in QuickNII (2017 CCFv3) [25]. Resulting.json anchoring files were imported to VisuAlign (v0.8 RRID:SCR_017978) for nonlinear refinement. Cell detections and VisuAlign outputs were imported into Nutil [26]. Brain regions of interest were defined using a custom Excel sheet. Object reports were generated with a minimum size of 4 pixels, point cloud density of 4, without object splitting, and extracting all coordinates. A custom MATLAB script (R2022b, MathWorks, Natick, MA, USA) re-formatted Nutil.csv outputs for statistical analysis and graphical representation in GraphPad Prism (v.9.3.1, San Diego, CA, USA).

### In vitro patch-clamp electrophysiology

In vitro electrophysiological experiments were done on a genetically modified strain (Tg(Drd1a-Cre)FK164Gsat/Mmucd), that selectively expresses Cre-recombinase in a subpopulation of layer 6 neurons across the entire cortical mantle. This L6-specific Cre line is crossed with a td-Tomato reporter line (Ai14). 18 mice aged 22.83 ± 6.03 were used for single-cell patch-clamp electrophysiological recordings (for details see Supplementary Table 1). An average of two L6 neurons were recorded per animal. Animals were anesthetized using 4% isoflurane followed by decapitation, and the brains were extracted in cold sucrose solution (40 mM NaCl, 3 mM KCl, 7.4 mM MgSO_4_.7H_2_O, 150 mM sucrose, 1 mM CaCl_2_, 1.25 mM NaH_2_PO_4_, 25 mM NaHCO_3_, and 15 mM glucose; osmolality 300 ± 10 mOsmol/kg).

Coronal cortical slices (250–300 μm thick) were cut using a vibratome (Leica VT1200S) and placed in an interface chamber containing artificial cerebrospinal fluid (aCSF) (126 mM NaCl, 3.5 mM KCl, 2 mM MgSO_4_.7H_2_O, 1.25 mM NaH_2_PO_4_, 24 mM NaHCO_3_, 2 mM CaCl_2_, and 10 mM glucose; osmolality 300 ± 10 mOsmol/kg) for the duration of the experiment. All solutions were bubbled with carbogen gas (95% O2/ 5% CO2). Whole-cell current-clamp recordings of mPFC neurons were performed in a submerged chamber ( ~ 32 °C) using borosilicate glass pipettes (5–12 MΩ) filled with internal solution (110 mM K-gluconate, 40 mM HEPES, 2 mM ATP-Mg, 0.3 mM GTP-NaCl, 4 mM NaCl, 3–4 mg/ml biocytin; pH ∼7.2; osmolality 270–290 mOsmol/kg). Electrical signals were amplified with a Multiclamp 700 B amplifier (Molecular Devices, Foster City, CA), low-pass filtered at 10 kHz and digitized at 20 kHz using a Digidata 1440 A (Molecular Devices). Hyperpolarizing and depolarizing square current pulses were applied in order to quantify intrinsic properties of the recorded neuron, with these step protocols performed at baseline and 2, 5 and 10 min after application of 200 nM YNT-185. The recordings were extracted and processed using Igor Pro 6 (Wavemetrics) and later analysed with custom-made Python scripts. All statistics were done using GraphPad Prism 10.

### In vivo injections

In vivo Orexin-B injections were performed on the same genetically modified strain of *Tg(Drd1a-Cre)FK164Gsat/Mmucd* used in the patch-clamp experiments. A total of 9 animals were used, aged 78.80 ± 31.71 days. The mice were anaethetised for the duration of the experiment using 2% isoflurane/medical oxygen mixture, implanted with a 16-channel linear electrode (Neuronexus) targeting mPFC (1.94 mm antero-posterior, 0.4 mm lateral and 1.75 mm dorso-ventral), with extracellular voltage signals acquired at 30 kHz using an RHD2164 64-channel amplifier board connected to an RHD2000 USB interface board and controlled by RHX Data Acquisition software (Intan Technologies). The animals were injected with 1–2 μL of either a 0.345 mM Orexin-B solution or a 1–2 µL saline solution in the left lateral ventricle (−0.1 mm antero-posterior, 0.75 mm lateral and: 1.90 mm dorso-ventral). A 15-min recording before, and a 20-min recording after the injection were performed in all animals. The first 5 min of the recording following the Orexin-B administration were not used for the analysis. Recordings were extracted and processed with custom-made Python scripts and all statistics were calculated with GraphPad Prism 10.

### Multielectrode electrophysiology

The network effect of silencing the L6-Drd1a population was evaluated in a Drd1a-Cre^+/-^:Ai14:Snap25^fl/fl^ genetically modified strain, and compared with Cre negative controls Drd1a-Cre^-/-^:Ai14:Snap25^fl/fl^. These Drd1a-Cre^+/-^:Ai14:Snap25^fl/fl^ animals express Td-Tomato and have Snap25 ablated in the Drd1a positive neurons, leading to severe reductions in evoked synaptic vesicle release from these neurons [27]. As an additional control, a similar genetical modification was performed in Rbp4-Cre:Ai14:Snap25^fl/fl^ which mirrors the Snap25 ablation of the previous population, but in Rbp4-Cre positive neurons [28].

9 adult mice aged 76.14 ± 5.03 were used. Brains from the animals were retrieved and the tissue was sliced using the same protocol as the one described for the in vitro whole-cell current-clamp experiments. An average of 3 slices per animal were used for each group. All experiments were performed by experimenters blind to the genotypes of the animals. Recordings were done using a high-density microelectrode array (MaxOne) from Maxwell Biosystems (Zürich, Switzerland). The MEA provides 26,400 electrodes in a 3.85 × 2.10 mm^2^ large sensing area with an electrode pitch of 17.5 μm [29]. A combination of up to 1024 electrodes can be recorded from simultaneously, with a sampling rate of 20 kHz. All recordings were 5 min long, each condition was done by triplicate. Baseline recordings were done using a continuous administration of 200 µM 4-AP in aCSF for 20 min before recordings started. Afterwards, the solution was changed for a 200 nM YNT-185 and 200 µM 4-AP in aCSF, following the same protocol as before. The recordings were extracted and pre-processed using custom-made Matlab and Python scripts. All statistics were done using GraphPad Prism 10.

### Behavioural experiments

The behavioural evaluation was performed using the same genetically modified strain as used for the high-density multielectrode electrophysiology: Drd1a-Cre^+/-^:Ai14:Snap25^fl/fl^ versus Drd1a-Cre^-/-^:Ai14:Snap25^fl/fl^. Anxiety-related behaviour was assessed using two protocols: i) the elevated plus maze (EPM), which consisted of an elevated platform at 50 cm from the floor and composed of four arms 30 cm long and 5 cm wide, and a 5 by 5 cm central zone. The arms were divided into two pairs, one pair of them enclosed by a 30 cm lateral wall and the other pair open. For this test, animals were placed in the central zone facing one of the open arms and allowed to explore the arms freely. Time spent in open arms and number of entries into open arms were recorded for 5 min using AnyMaze tracking system (Stoelting). ii) the light-Dark box (LDB), which consisted of a 44 by 21 by 21 cm cage divided into light and dark chambers by a 13 cm long and 5 cm high opening. One area was made dark with black spray paint, covered from the external environment, and maintained without illumination. The other compartment was open and brightly lit. The lit area corresponded to 2/3 of the total area of the box. Measurement of spontaneous locomotor activity (LMA) in a novel environment was conducted in plexiglass cages (20 × 35 cm), with movement detected with an infrared photobeams system (PAS-Open Field, San Diego Instruments) over the course of 2 h every minute. All behavioural experiments were performed by experimenters blind to the genotypes of the animals.

## Results

**Fig. 1:**
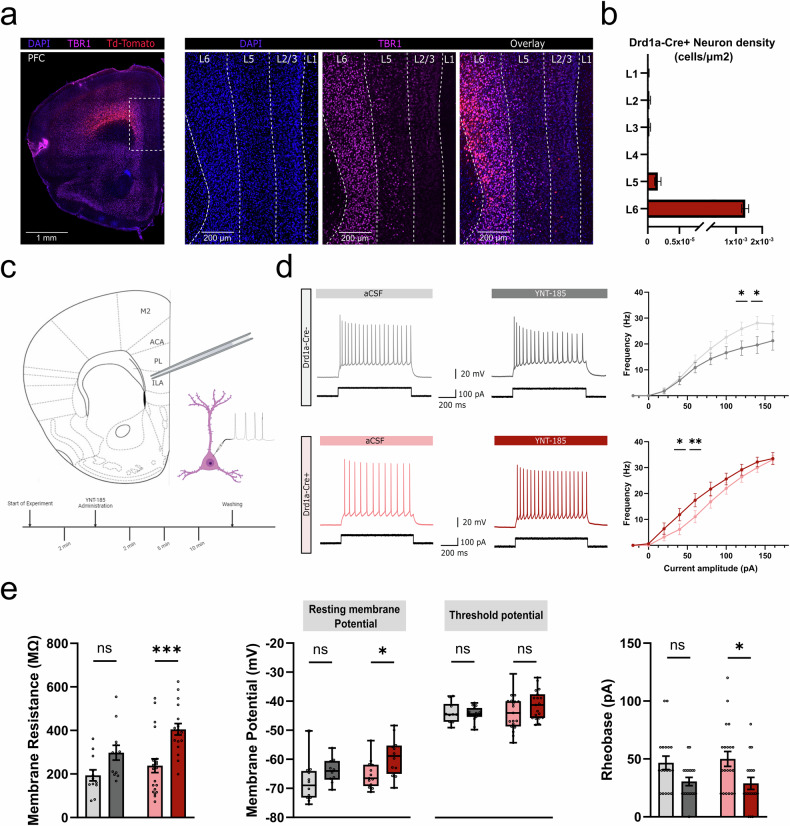
Drd1a-Cre+ neurons in layer 6 selectively respond to Orexin agonists in mPFC. **a** Representative image of DAPI & TBR1 immunostained sections of Drd1a-Cre:Ai14 mice. These animals express Td-Tomato in the Drd1a-Cre+ neurons. Right panel shows a zoomed image of the medial prefrontal cortex (mPFC). DAPI staining was used to set the layer borders and TBR1 staining was used to delineate layer 6. **b** Quantification of the number of cells per layer shows that Drd1a-Cre+ neurons are found almost exclusively in layer 6 of the mPFC (n = 5). **c** Schematic of the patch-clamp protocol. Drd1a-Cre+ neurons in the infralimbic cortex (ILA) were targeted for whole-cell current-clamp recordings and intrinsic properties were measured at baseline and at the 2-, 5- and 10-min following application of 200 nM YNT-185. **d** Example voltage recording after 100 pA stimulation in Drd1a-Cre- (gray) and Drd1a-Cre+ (red) neurons before and after the administration of YNT-185. Input-output curve shows the changes in spiking activity after increasing stimulation during baseline (aCSF) and administration of YNT-185. Drd1a-Cre- neurons (gray) become significantly inhibited at higher amplitudes (n = 12; p = 0.0199; Mixed effect analysis with Holm-Šídák’s multiple comparisons). Drd1a-Cre+ neurons (red) respond significantly higher at lower amplitudes after YNT-185 administration but plateau at higher amplitudes (n = 12; p = 0.0034; Mixed effect analysis with Holm-Šídák’s multiple comparisons). **e** Membrane properties show significant changes after YNT-185 administration. Rheobase measurements show a reduction in the stimulus required to elicit an AP (n = 18; p = 0.0134; Two-way ANOVA with Šídák’s multiple comparisons correction). All numbers were reported as the mean with SEM. Error bars represent SEM, each datapoint is represented as a dot. All data presented as mean ± SEM, *p < 0.05, **p < 0.01, ***p < 0.001, ****p < 0.0001.

We first explored the anatomical distribution of Drd1a-Cre+ neurons in mPFC of Drd1a-Cre:Ai14 mice, using DAPI staining to determine the layer borders with T-Box brain 1 (TBR1) co-staining used to confirm L6 (Fig. 1a) and complexin-3 (Cplx3) co-staining to identify L6b (Figure S1). We found that Drd1a-Cre+ neurons can be found almost exclusively in L6a and L6b of the mPFC (98.79%) (Fig. 1b).

The mPFC includes infralimbic (ILA), prelimbic (PL) and anterior cingulate areas (ACA). In order to explore the orexin-sensitivity of L6 mPFC neurons, we performed whole-cell current-clamp recordings in the ILA of acute brain slices of Drd1a-Cre:Ai14 mice. In recordings from Drd1a-Cre+ L6 neurons, the application of a 200 nM solution of OX2R agonist, YNT-185, increased intrinsic excitability due to an increase in membrane resistance and depolarisation of resting membrane potential (Fig. 1c–e). These changes resulted in a reduced rheobase current, without a change in the voltage threshold for action potential generation (Figure S2).

The increased spike rate for a given current step was associated with an increase in the half-width and a decrease in the spike amplitude, consistent with previous reports of frequency-dependent changes in action potential waveform [30–33] (Figure S3). Recordings from Drd1a-Cre- L6 neurons showed that YNT-185 only reduced the spike frequency at higher current steps (Fig. 1c–g). Recent single-cell transcription factor studies of layer 6 neurons have shown that subclasses of both excitatory and inhibitory L6 neurons can express orexin receptors [34], and thus the reduction in Drd1a-Cre- neuron firing rate could be due to the activation of L6 inhibitory neurons.

Both groups of recorded neurons (control and Drd1a-Cre+) were labelled with biocytin for post hoc neuronal identification and morphological reconstruction. All neurons were located in layer 6 of the mPFC. Although most recorded cells were found in layer 6b (66%), no clear difference between these and cells in layer 6a was observed in terms of their morphology or physiological behaviour (Figure S4a). Recorded neurons were reconstructed and grouped according to genotype. Morphological reconstruction of the recorded neurons showed that there was no significant difference between control and Drd1a-Cre+ neurons (Figure S4b). Moreover, all recorded neurons were morphologically identified as excitatory neurons based on the shape and distribution of their axodendritic projections [35]. While Drd1a-Cre+ L6 neurons may not be uniquely sensitive to orexin in mPFC, these results are consistent with a role for Drd1a-Cre+ L6 neurons in mediating orexin-induced increases in cortical excitability across sensorimotor cortex and mPFC.

**Fig. 2:**
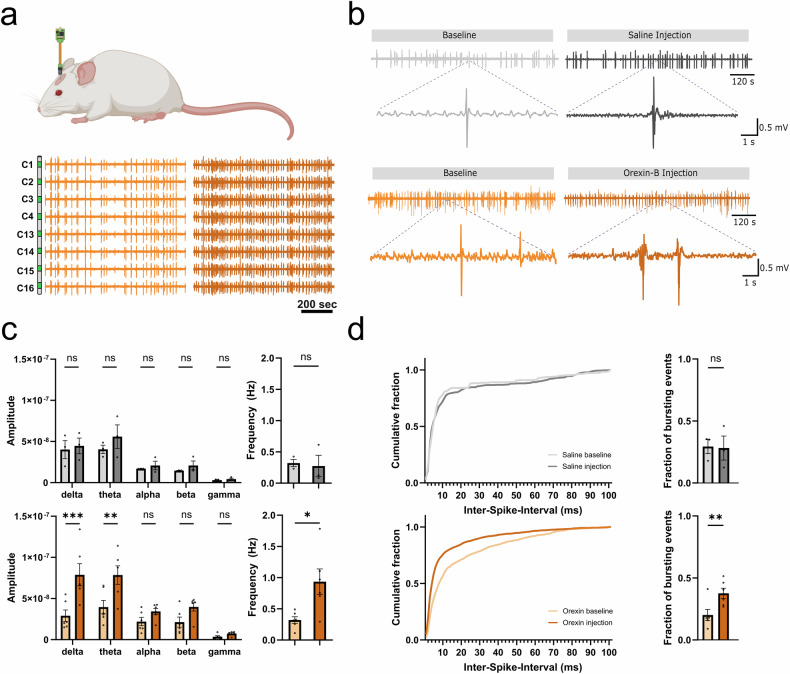
Intraventricular administration of orexin increases mPFC network activity in vivo*.* **a** Schematic of the position of the linear electrode in the mPFC. This configuration allows for a linear electrode to record throughout the mPFC. From dorsal to ventral the recordings correspond to the supplementary motor cortex (M2), anterior cingulate cortex (ACA), prelimbic cortex (PL), and infralimbic cortex (ILA). The spacing between the recording sites (green) is 110 µm. **b** Example of recordings of an electrode in ILA through the experiment before and after the injection of saline (top) or orexin-B (bottom). **c** Power spectral density (PSD) analysis of the recordings for the control (top) and the orexin experiments (bottom) in the ILA. No significant changes were observed for controls. Orexin administration significantly increased the spectral power measured in both delta and theta bands (n = 6; p = 0.0001 & p = 0.0024 respectively; Two-way ANOVA with Šídák’s multiple comparisons correction). This reflected an increase in burst frequency after the orexin injection compared to baseline and the saline injections (n = 6; p = 0.0308; Two-tailed paired t-test). **d** Orexin-B intraventricular injections increase the number of evoked bursting events. Compared to a saline control (top), Orexin-B injections increased the proportion of events with an inter-spike-interval smaller than 50 ms. The increase in the number of bursting events was significant after the orexin-B injection. The increase in evoked spiking activity was not reflected as an increment in bursting activity for any of the injections (n = 6; p = 0.0088; Mixed effect analysis with Šídák’s multiple comparisons corrections). All numbers were reported as the mean, error bars represent SEM. All data presented as mean ± SEM, *p < 0.05, **p < 0.01, ***p < 0.001, ****p < 0.0001.

While activation of OX2R increased the intrinsic excitability of Drd1a-Cre+ mPFC L6 neurons, it did not lead to sustained spiking activity as observed with orexin application in the sensory cortex [17]. To examine whether these changes in intrinsic excitability might be sufficient to activate cortical circuits, we performed 16-channel linear electrode recordings of the local field potential (LFP) in lower layers of mPFC of naïve mice under isoflurane anaesthesia (Fig. 2a). Intraventricular injections of 1–2 µL 0.345 mM Orexin-B solution increased the burst frequency primarily at low-frequency power, both delta (0.5–4 Hz) and theta (4–8 Hz) frequency bands, but had no effect on higher frequencies [36] (Fig. 2c, d). These effects were due to an increase in the occurrence of delta waves. This was also associated with an increase in the clustering of bursting events as has been seen for other neurotransmitters in the mPFC, such as acetylcholine [37] and dopamine [38, 39] (Figure S4). No changes in network activity were observed following intraventricular injections of 1–2 µL of saline solution (Fig. 2c, d).

**Fig. 3:**
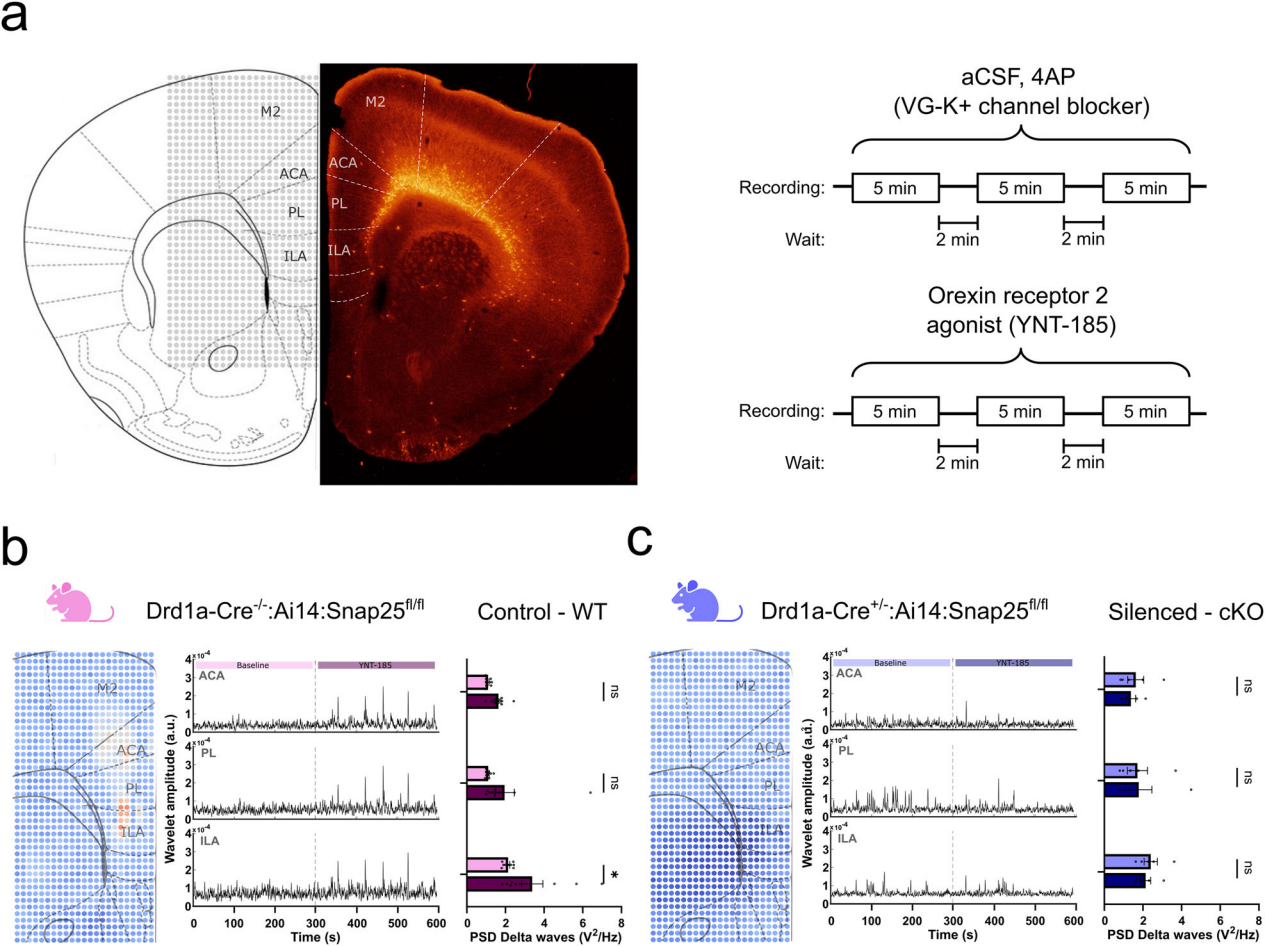
Chronic genetic silencing of Drd1a-Cre+ neurons abolish orexin response in mPFC. **a** Schematic of the positioning of the MEA in the brain slice. The position allows for the simultaneous recording of the ILA, PL and ACA areas. Right panel shows the drug administration protocol for baseline (top) and YNT-185 administration (bottom). **b** PSD analysis of the network response to application of an ORX2 agonist, 200 nM YNT-815, in the control animals (Drd1a-Cre^-/-^:Ai14:Snap25^fl/fl^). Each dot represents an electrode in the array and warmer colours represent increases in delta power after the drug administration. An increase in activity throughout mPFC can be seen. Wavelet transform analysis shows the change in delta power across time before and after YNT-185 administration. Right panel shows the changes in delta power per area. All areas show increased activity, which is significant in the ILA (n = 9; p = 0.0305; Mixed effect analysis with Šídák’s multiple comparisons corrections). **c** The same experiments were repeated in Drd1a-Cre-silenced animals (Drd1a-Cre^+/-^:Ai14:Snap25^fl/fl^). There was a lack of YNT-185-driven cortical activation when these neurons were silenced. Wavelet transform analysis confirms the lack of increase in delta activity after the YNT-185 administration. All comparisons were done using mixed-effect Two-Way ANOVA analysis with Šidák correction. All numbers were reported as the mean with SEM. Error bars represent SEM, each datapoint is represented as a dot. All data presented as mean ± SEM, *p < 0.05, **p < 0.01, ***p < 0.001, ****p < 0.0001.

To confirm that orexin-induced increases in delta activity were due to direct effects in mPFC, rather than indirect effects on subcortical arousal systems, we used high-density planar multielectrode arrays to record network bursting activity in acute slices of mPFC induced by the application of 200 µM 4-AP. These bursts could be observed in the LFP, with power concentrated in the delta frequency range, and additional application of 200 nM YNT-185 increased both delta power and frequency of events (Fig. 3b), as observed with orexinergic activation in vivo. To determine whether these effects were mediated by Drd1a-Cre+ L6 neurons, we repeated the experiments with mice in which these neurons had Snap25 ablated (Drd1a-Cre^+/-^:Ai14:Snap25^fl/fl^), thus eliminating action potential evoked release [27]. Functional silencing of Drd1a-Cre+ L6 neurons occluded the effects of YNT-185 (Fig. 3c). This was not due to a simple decrease in the overall levels of synaptic activity, as YNT-185 increased activity throughout the mPFC in brain slices from mice in which layer 5 Rbp4-Cre+ neurons had been silenced (Figure S5). This suggests that Drd1a-Cre+ neurons selectively gate the orexinergic activation of mPFC.

**Fig. 4:**
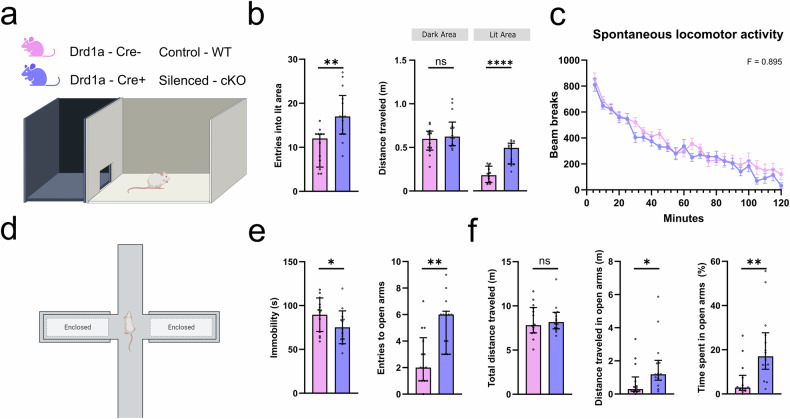
Anxious behaviour is altered after chronic manipulation of the cortical Drd1a-Cre+ neurons. **a** Schematic of a Light-Dark Box (LDB) experimental protocol. On top, the colour coding for genotype of the control (Drd1a-Cre^-/-^:Ai14:Snap25^fl/fl^; WT) and Drd1a-Cre-silenced mice (Drd1a-Cre^+/-^:Ai14:Snap25^fl/fl^; cKO). **b** cKO animals show an increase in entries into (n = 12; p = 0.0011; Mann Whitney test) and total movement inside the lit area (n = 12; p < 0.0001 ; Mann Whitney test). **c** Comparison of spontaneous locomotor activity in novel, photocell locomotor activity cages between WT and cKO animals shows no difference in the total movement of these animals **d** Schematic set-up for the Elevated Plus Maze (EPM). **e** cKO animals show a reduction in immobility after being placed in the EPM (n = 14; p = 0.0444; Mann Whitney test), as well as increased entries into the open arms (n = 14; p = 0.0016; Mann Whitney test). **f** Total distance travelled was similar between genotypes, but both the distance moved in open arms (n = 14; p = 0.0141; Mann Whitney test), and the overall time spent in them (n = 14; p = 0.0012; Mann Whitney test) was significantly increased. All behavioural comparisons were tested by using Mann-Whitney nonparametric tests and all numbers were reported as the median. Error bars represent interquartile ranges, each datapoint is represented as a dot. All data presented as mean ± SEM, *p < 0.05, **p < 0.01, ***p < 0.001, **** *p* < 0.0001.

The mPFC plays a role in anxiety and aversion (Heidbreder & Groenewegen, 2003) as well as learning and memory tasks (Morgan & LeDoux, 1995). In order to explore whether Drd1a-Cre+ L6 neurons play a role in regulating anxiety, we compared the behaviour of mice with chronic silencing of Drd1a-Cre+ L6 neurons (Drd1a-Cre^+/-^:Ai14:Snap25^fl/fl^) with littermate controls (Drd1a-Cre^-/-^:Ai14:Snap25^fl/fl^) in two behavioural paradigms used to assess anxiety phenotypes: the Light-Dark Box (LDB) protocol [40] and the Elevated Plus Maze (EPM) [41, 42]. In the LDB, animals tend to avoid the lit areas since they are a source of potential danger. Drd1a-Cre:silenced (cKO) mice showed significant increases in the entries into the lit areas, as well as the total distance travelled in this area (Fig. 4a, b). Importantly, the distance travelled in the dark area remained similar across genotypes. Furthermore, assessment of spontaneous locomotor activity levels in novel photocell activity cages revealed no differences between the genotypes, demonstrating that the cKO mice were not hyperactive per se (Fig. 4c). Like the previous protocol, in the EPM, animals have a choice as to whether to explore the exposed, open arms or remain in the enclosed arms (Fig. 4d). Consistent with a reduced anxiety phenotype, cKO animals showed a reduction in immobility time and an increase in the number of entries to these open arms (Fig. 4e). Furthermore, these animals showed an increase in the distance moved and the time spent in the open arms, while the total amount of movement remained similar to the control animals (Fig. 4f).

## Discussion

In this study, we provide evidence that orexin and OX2R agonists can directly activate prefrontal networks in adult mice, and that this orexinergic neuromodulation is dependent on L6 Drd1a-Cre+ neurons. While orexinergic activation was observed in the entire mPFC, it was particularly focused on the infralimbic area, an area known for its role in anxiety behaviour [36, 43–45]. Indeed, silencing Drd1a-Cre+ neurons led to a reduction in anxiety-related behaviours.

A previous study has shown that L6 Drd1a-Cre+ neurons in the somatosensory cortex strongly respond to orexin (Zolnik et al., 2023). Here, we used the same genetically-modified Cre-line [13] to target whole-cell patch-clamp recordings from Drd1a-Cre- and Drd1a-Cre+ L6 neurons in brain slices of mPFC and tested the effects of a selective OX2R-agonist, YNT-185 [46]. In Drd1a-Cre+ neurons, YNT-185 produced a significant membrane potential depolarisation, associated with a decrease in rheobase current and increased excitability, consistent with previous reports of the effect of orexin on neuronal activation [47]. While Drd1a-Cre+ neurons in somatosensory cortex are concentrated in L6b, Drd1a-Cre+ neurons in mPFC are spread across L6a and L6b, but these results suggest that Drd1a-Cre+ neurons may represent a functionally homogenous population a deep layer cortical neuron.

The application of YNT-185 did not produce a significant excitatory effect on Drd1a-Cre- L6 neurons in mPFC, but did appear to reduce the spiking responses to larger amplitude current steps, compared to both baseline and neighbouring Drd1a-Cre+ neurons. This could be a consequence of the activation of deep layer inhibitory neurons in the cortex, which have been reported to express orexin receptor transcripts [34]. Such orexinergic excitation of GABAergic neuron populations has previously been reported to contribute to the regulation of feeding and homeostasis via the arcuate nucleus of the hypothalamus [48] as well as modulation of aggressive behaviour via the lateral habenula [49].

To understand how these effects of orexin receptor activation might impact mPFC network activity in vivo, we tested the effects of intraventricular administration of orexin-B using multielectrode recordings from the deep layers of mPFC in anaesthetised mice. Under these conditions, cortical networks show slow bursting activity, with LFP power concentrated in the delta- and theta-frequency ranges, and we found that orexin-B increased the power in these frequency bands and the incidence of bursting events. This is consistent with orexin having an excitatory effect on mPFC networks, although it is less clear how the changes in delta/theta activity relate to the orexinergic modulation of mPFC dynamics in unanaesthetised mice. However, it has been shown that an increase in power at lower frequencies (delta and theta) is the most dominant electrophysiological signature in conditions such as anxiety, ADHD, OCD, and schizophrenia [50–52], so understanding how such activity is regulated by the orexinergic system could have relevance to a range of neurological and psychiatric conditions [53–55].

In order to examine whether Drd1a-Cre+ neurons are necessary for orexinergic activation of the mPFC, we examined mice in which this subpopulation of neurons had been silenced using the conditional knockout of SNAP25. Blocking evoked synaptic release in Drd1a-Cre+ neurons from birth might be expected to affect cortical development, by preventing the relay of local and long-range inputs, including those from the hypothalamus. Indeed, hypothalamic agouti-related peptide-expressing neurons, which also receive direct orexinergic input [8, 56], have been shown to be important for the development of cellular and functional integrity in mouse PFC [8, 56]. However, cortical development appears normal in Drd1a-silenced animals [27].

We examined the differences in orexin-sensitivity across the mPFC of wild-type and knockout mice using coronal brain slices mounted on a large area (3.85 × 2.10 mm^2^) high-density multielectrode arrays [29]. In wild-type mice, consistent with our previous observations, YNT-185 administration evoked a significant increase in the network activity in mPFC, especially in the ILA. Nevertheless, in the Drd1a-silenced animals, the administration of orexin agonists produced no significant increase in cortical activation. Importantly, the ILA has been reported as a key modulator of anxiety and stress behaviours [57, 58], wherein ILA hyperexcitability produces anxiety-like behaviours in otherwise healthy adult mice [45]. We repeated these experiments in an Rbp4-silenced line, in which evoked synaptic release is blocked in a subpopulation of L5 pyramidal neurons. We observed that the administration of YNT-185 increased activity throughout the mPFC (Figure S5). Thus, we confirmed that the disruption of the network activity was exclusive to the manipulation of the Drd1a-Cre+ population, supporting the notion that these neurons gate orexinergic activation of prefrontal networks.

At the behavioural level, we found that chronically silencing the Drd1a-Cre+ neuronal population had significant effects in two known anxiety tests: A Light-dark box (LDB) and an elevated plus maze (EPM). Compared to controls, the Drd1a-silenced animals spent more time and moved more freely in both the brightly lit areas of the LDB and the open arms of the EPM [40, 42, 59]. Both these results suggest a reduction in anxious behaviour, which is more likely to reflect changes in higher association areas, including mPFC, rather than sensorimotor cortex. These Drd1a-Cre+ L6 neurons may thus provide a promising new target for the pharmacological treatment of anxiety disorders.

## Supplementary information

Supplementary table

Supplementary figure

## Data Availability

All data analysed and corresponding scripts used in this study are available upon request from the corresponding authors (ZM & EM).
