# Supplementary material for: An orexin-sensitive subpopulation of layer 6 neurons regulates cortical excitability and anxiety behaviour
Source: Transl Psychiatry. 2025 Apr 14;15:147. doi: 10.1038/s41398-025-03350-2 (PMC11997144; doi:10.1038/s41398-025-03350-2)
Supplement: Supplementary file 2 — Supplementary figure [file 41398_2025_3350_MOESM2_ESM.pdf]

Supplementary Information

Figure S1: Determination L6a & L6b

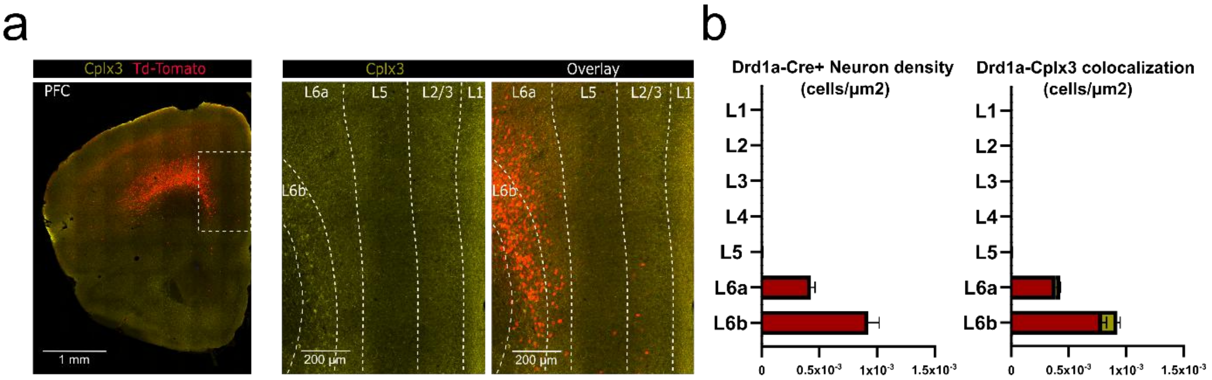

a) Representative image of Cplx-3 immunostained sections of Drd1a-Cre:Ai14 mice. These animals express Td-Tomato in the Drd1a-Cre+ neurons. Determination of the L6a-L6b border was done using Cplx3 staining (yellow). **b)** There is a higher density of Drd1a-Cre+ neurons in L6b, although is not exclusive to this layer. The proportion of Drd1a+/Cplx3+ neurons is higher in the deeper parts of L6.

Figure S2: Spiking activity in Drd1a-Cre+ and Drd1a-Cre- neurons in PFC layer 6

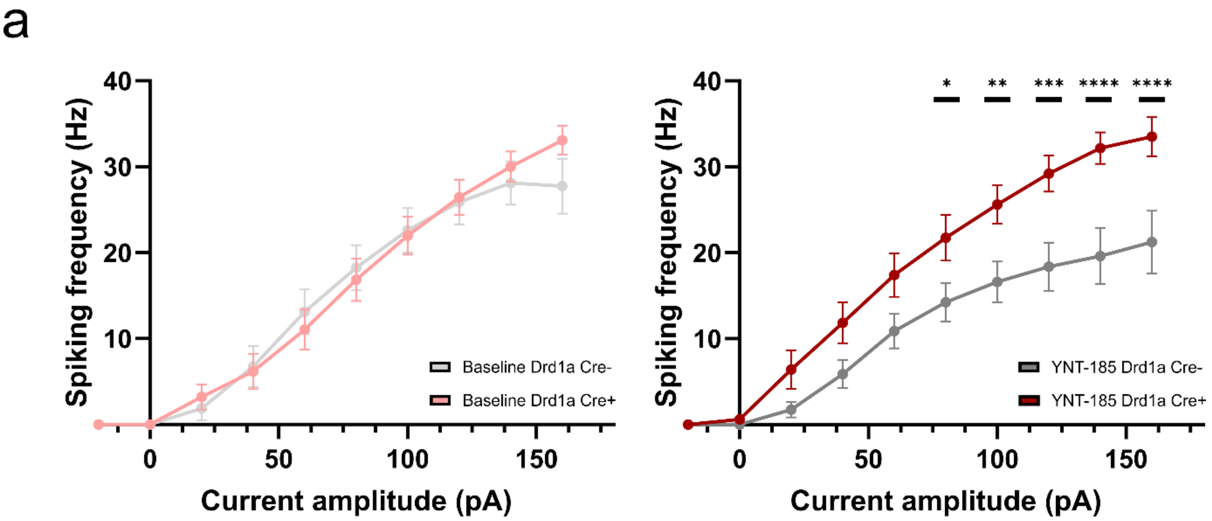

a) The input/output curve shows the changes in spiking activity after increasing stimulation during baseline (aCSF) (Left) and after administration of orexin agonists (YNT-185) (Right). Baseline activity shows no difference between distinct genotypes. Drd1a-Cre+ neurons respond significantly more to the administration of YNT-185 as compared to the Drd1a-Cre- neurons ($n = 15$; $p < 0.0001$; Mixed effect analysis with Šídák's multiple comparisons). All numbers were reported as the mean, error bars represent SEM.

Figure S3: Action potential properties of recorded Drd1a-Cre+ neurons

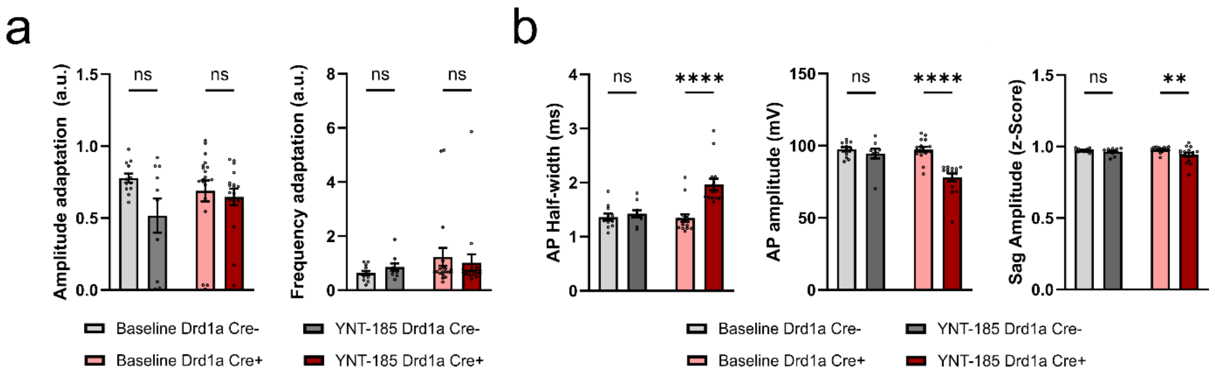

a) Administration of YNT-185 had no significant effect on the evoked action potential frequency or amplitude adaptation. We also observed no difference between Drd1a-Cre+ and Drd1a-Cre- neurons. **b)** Consistent with previous reports of frequency-dependent changes, action potentials showed a significant increase in the half-width and a decrease in the spike amplitude (n = 15; $p < 0.0001$; Mixed effect analysis with Šídák's multiple comparisons). All numbers were reported as the mean, error bars represent SEM.

Figure S4: Spiking activity in Drd1a-Cre+ and Drd1a-Cre- neurons in PFC layer 6

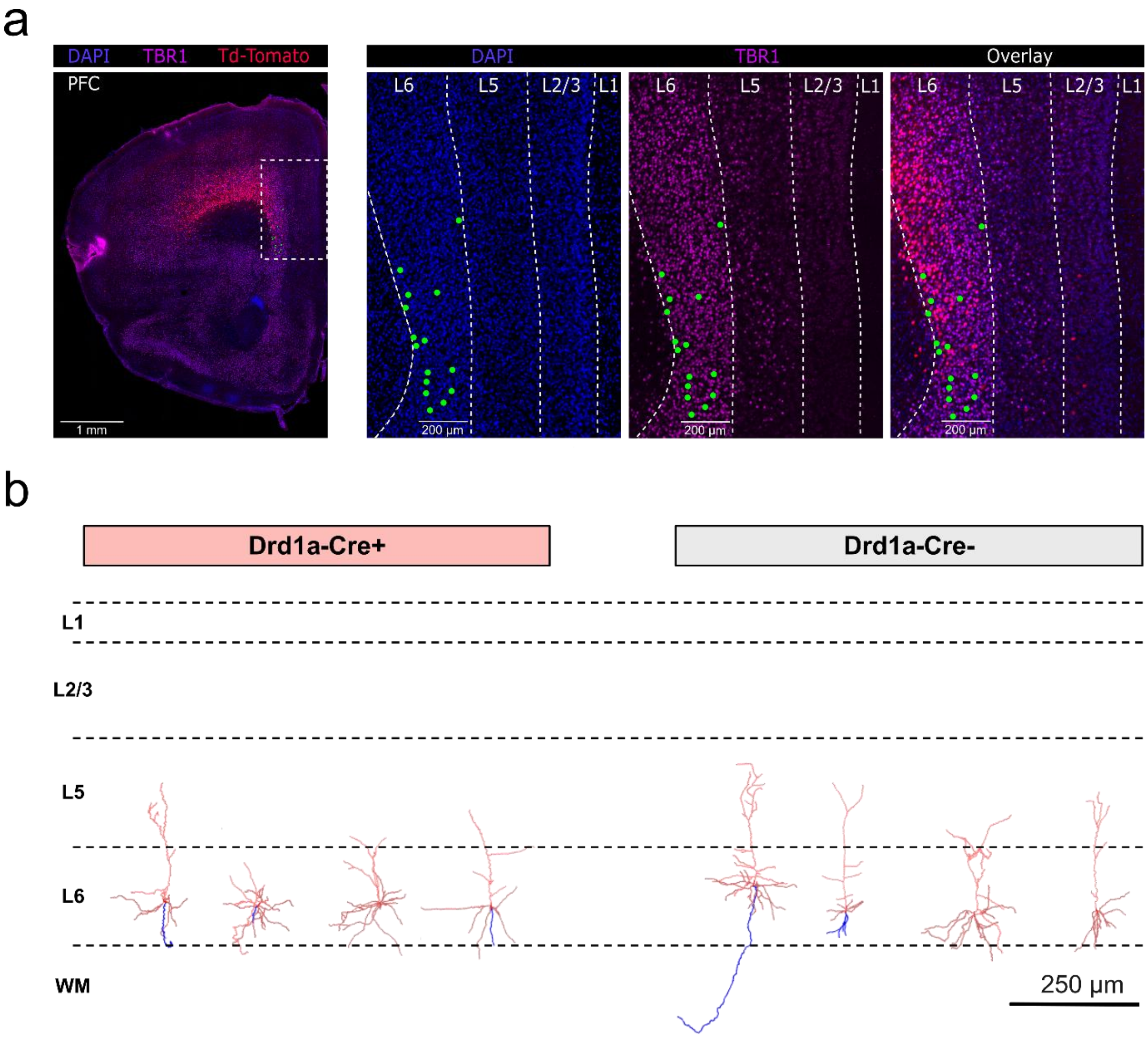

a) Representative image of DAPI &TBR1 immunostained sections of Drd1a-Cre:Ai14 mice. These animals express Td-Tomato in the Drd1a-Cre+ neurons. Right panel shows in green dots the location of the patch-clamp recorded neurons in the mPFC. **b)** Examples of morphological reconstructions of recorded neurons in layer 6. Both subpopulations have morphologies consistent with excitatory neurons in deep layers (Yen et al., 1985)

Figure S5: Multielectrode activation of L5 silenced animals

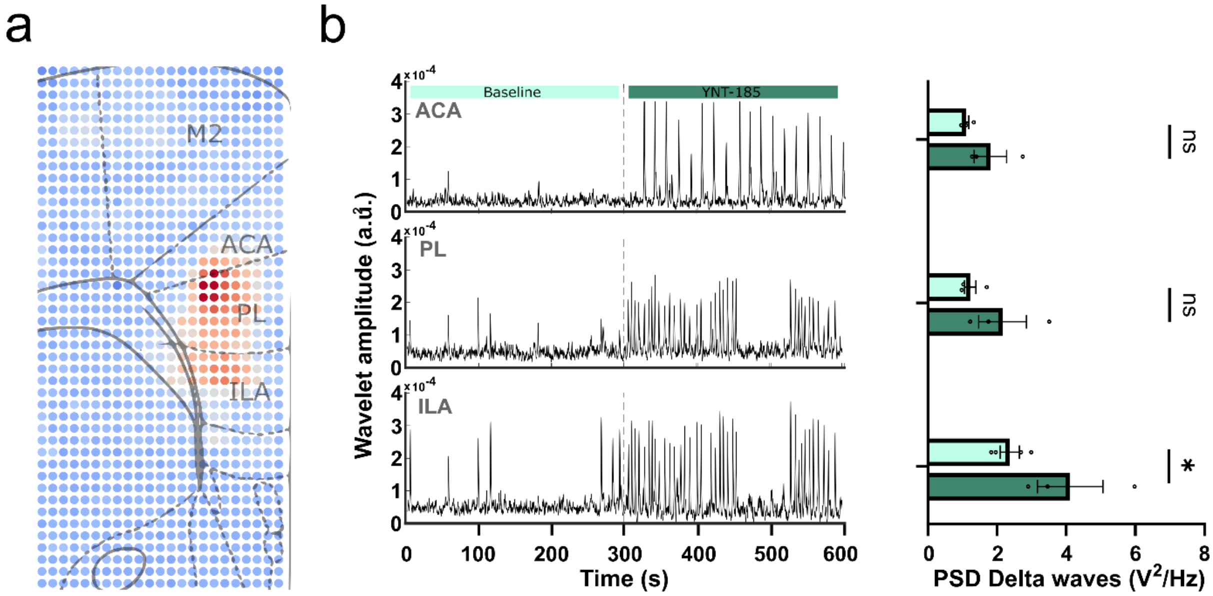

a) PSD analysis of the network response to Orexin agonist in the layer 5 silenced animals (Rbp4-Cre+/+:Snap25fl/fl). The administration of orexin significantly increases the delta activity in mPFC. The silencing of layer 5 had no effect in the orexinergic activation and, together with the findings of Figure 4, demonstrates that the orexinergic activation of the neocortex depends on the activation of layer 6 Drd1a-Cre+ neurons. **b)** Wavelet transform of the recordings shows an increase in activity across time in all mPFC areas after YNT-185 administration. All comparisons were done using Mixed-effect Two-Way ANOVA with Šidák correction. All numbers were reported as the mean with SEM. Error bars represent SEM, each datapoint is represented as a dot
